# pH-Responsive chromogenic-sensing molecule based on bis(indolyl)methene for the highly selective recognition of aspartate and glutamate

**DOI:** 10.3762/bjoc.7.29

**Published:** 2011-02-16

**Authors:** Litao Wang, Xiaoming He, Yong Guo, Jian Xu, Shijun Shao

**Affiliations:** 1 Key Laboratory of Chemistry of Northwestern Plant Resources and Key Laboratory for Natural Medicine of Gansu Province, Lanzhou Institute of Chemical Physics, Chinese Academy of Sciences, Lanzhou 730000, P. R. China; 2 Graduate School of the Chinese Academy of Sciences, Beijing 100039, P. R. China

**Keywords:** aspartate and glutamate, bis(indolyl)methene, colorimetric sensor, molecular recognition, proton transfer

## Abstract

Bis(indolyl)methene displays high selectivity and sensitivity for aspartate and glutamate in water-containing medium based on the proton transfer signaling mode. The presence of acid can easily induce proton transfer to the basic H-bond acceptor moiety, which modulates the internal charge transfer state of the bis(indolyl)methene skeleton and gives rise to dramatic change in color. The detection limits for aspartate and glutamate were 0.80 ppm and 1.12 ppm, respectively.

## Introduction

**Figure 1:**
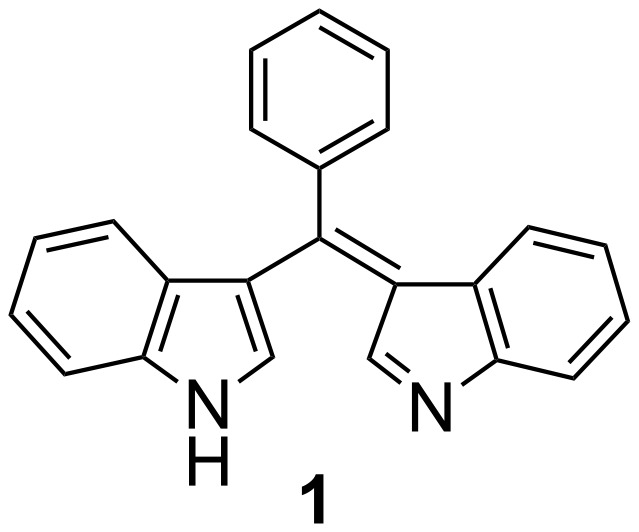
Structure of sensor **1**.

The development of artificial receptors for the selective recognition of biologically important species has attracted much attention [1–2]. However, compared to the large number of chromo/fluororeceptors for cations or anions [3–7], the development of artificial receptors for amino acids is quite limited. The effective and selective molecular recognition or sensing of unprotected amino acids in aqueous solution is still a challenging problem due to their highly hydrophilic character [8]. Several studies have shown that aspartate (Asp) and glutamate (Glu) are important neurotransmitters [9–11], thus the recognition or sensing of these amino acids by synthetic receptor molecules is of great interest [12–14]. The bis(indolyl)methene molecule **1** (Figure 1), possessing an acidic H-bond donor moiety and a basic H-bond acceptor moiety, could act not only as a color-reporting group but also as a binding affinity control group. The anion sensing properties of **1** based on acidic H-bond donor moiety have been studied previously in our laboratory [15]. The strong hydrogen bonding to, or protonation/deprotonation of, the indolyl moiety modulate the internal charge transfer (ICT) state of bis(indolyl)methene and give rise to large color changes. In aqueous solution, the effective recognition based on strong hydrogen bonding is hardly achieved because of the strong hydration to hydrogen bonding sites of both receptor and guest. However, the basic H-bond acceptor moiety of **1** is very sensitive to changes of pH. Herein, we report a pH-responsive chromogenic-sensing molecule **1** that shows high selectivity for Asp and Glu in a mixed acetonitrile/water medium.

## Results and Discussion

**Figure 2:**
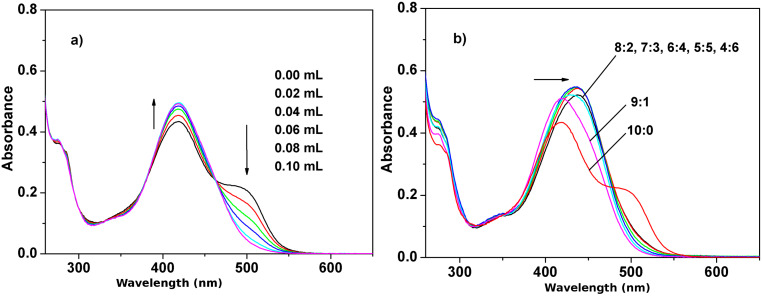
Changes in UV–vis spectra of **1** (5.0 × 10^−5^ M) after addition of: (a) 0–0.10 mL H_2_O; (b) various ratios of the CH_3_CN/H_2_O (fixed volume is 4 mL).

The synthesis of **1** was according to previously described methods [15]. In acetonitrile receptor **1** produces a yellow solution and displays two strong absorption bands at 277 and 423 nm in acetonitrile (Figure 2a). The strong absorption band at 423 nm (hence the yellow color of the solution) can be assigned to π–π* transitions of the conjugated bisindole skeleton. A less strong shoulder peak at 500 nm is related to its intermolecular hydrogen bonding interaction and disappears on the addition of a small quantity of a polar protic solvent such as CH_3_OH or H_2_O to the solution. In the presence of 0–0.10 mL H_2_O, the intensity of the band at 423 nm increases slightly, while the shoulder at 500 nm disappears, which suggests that H_2_O disturbs the intermolecular hydrogen bond assembly of **1** itself. On further increasing the water content according to various ratios of the acetonitrile/water (total volume = 4 mL), the band at 423 nm of receptor **1** shifts to 435 nm as a result of the solvent effect (Figure 2b). Moreover, the absorption band at 435 nm decreases when the ratio of the acetonitrile to water reaches 4:6 (v/v), which is probably related to the solubility of **1**. As a result, the amino acid recognition and sensing properties of **1** were investigated in acetonitrile/water in a 1:1 (v/v) ratio.

**Figure 3:**
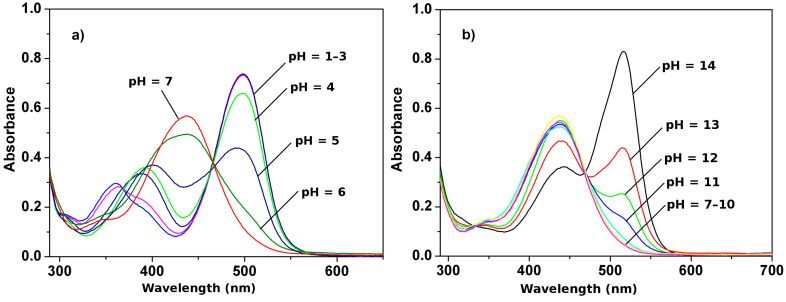
pH-dependent changes in UV–vis spectra of **1** (5.0 × 10^−5^ M) in CH_3_CN/H_2_O (1:1, v/v) at (a) pH = 1–7, and (b) pH = 7–14.

Next, the pH-dependent spectral properties of **1** were studied in acetonitrile/water. As shown in Figure 3a, when pH < 6 (AcOH and NaOH were used to adjust the pH values of the solutions), the notable spectral response of receptor **1** at 500 nm could be observed and almost reached a maximum at pH = 3. This is due to protonation of receptor **1** which is very sensitive to slight pH changes in aqueous acidic medium. However, in the pH 7–10 range, no obvious spectral changes of **1** were observed. With increasing pH of the solution (Figure 3b), a new absorption band appeared at 517 nm due to the deprotonation of **1** [15]. The processes taking place can be summarized as follows:


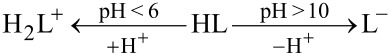


**Figure 4:**
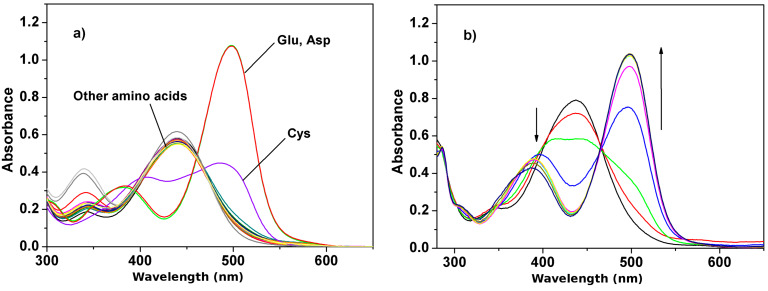
Changes in UV–vis spectra of **1** (5.0 × 10^−5^ M) in CH_3_CN/H_2_O (1:1, v/v) after addition of: (a) 25 equiv of Gly, Ala, Val, Leu, Iso, Phe, Thr, Glu, Asn, Met, Ser, Pro, Trp, Lys, Arg, His, Tyr, Glu, Asp and Cys; (b) 0, 1, 2, 3, 4, 5, 8, 10, 20, 50, 100 equiv of Asp.

We then made use of the sensitivity of receptor **1** towards slight changes in pH to investigate the sensing properties for 20 natural amino acids using UV–vis spectroscopic techniques. As shown in Figure 4a, upon the addition of 25 equiv of amino acids in acetonitrile/water (1:1, v/v), only Asp and Glu gave rise to perceptible spectral changes with the effect of changing the color of the solution instantly from yellow to red. This gives the potential for “naked eye” detection. With increasing concentration of Asp, the absorption band at 435 nm decreased accompanied with a blue-shift to 388 nm and at the same time a new strong absorption band at 500 nm continuously increased in intensity until it reached a maximum upon the addition of 5 equiv of Asp (Figure 4b),. This is responsible for the observed color change of the solution. A well-defined isobestic point at 466 nm suggests the formation of stable protonated complex and a simple one step equilibrium [16–17]. The titration spectra of the Glu almost resembled that of Asp. A similar, but less remarkable spectral change was observed upon addition of cysteine. On the other hand, no noticeable changes in color and absorption spectra were observed in the presence of various neutral amino acids and basic amino acids under the same conditions (even at much higher amino acids concentrations), which means that the receptor **1** exhibits negligible perturbation. The detection limits for Asp and Glu were determined to be 0.80 ppm and 1.12 ppm, respectively [18–19]. These results indicate that the receptor **1** has displayed high selectivity and sensitivity for Asp and Glu in water-containing medium.

The changes in color and absorption spectra may be ascribed to proton transfer to **1**. Because of the acidic characteristic of Asp (pI = 2.77) and Glu (pI = 3.22) [20], they are quite capable of protonating **1**, which modulates the internal charge transfer (ICT) and results in a drastic spectral change from 435 nm to 500 nm arising from the protonated receptor [H_2_L]^+^. For cysteine, due to its slight acidity compared to neutral and basic amino acids, it may also partly protonate **1**, however, the spectral change observed does not achieve the same level as the acidic amino acids even in the presence of 100 equiv of cysteine. On the other hand, compared with a strong base NaOH, the basicity of the basic amino acids (such as Arg or Lys) is not strong enough to induce deprotonation of **1**. These results show the high selectivity and sensitivity of **1** towards the acidic amino acids in aqueous medium.

The ratio of acetonitrile to water does not affect the sensing of amino acids. In a mixed solution (CH_3_CN/H_2_O, 3:1, v/v) of **1**, the addition of amino acids produced the similar effects. When ether was added to the red mixed solution (in CH_3_CN/H_2_O, 1:1) of **1** and Asp/Glu (containing 5.0 × 10^−5^ M **1** and 1.5 × 10^−3^ M Asp/Glu) and the mixture shaken carefully, the upper organic phase turned yellow, while the aqueous phase became colorless. This suggests that protonation is reversible.

## Conclusion

In conclusion, bis(indolyl)methene, containing a conjugated bisindole skeleton, provides an easy-to-make, simple and efficient chromogenic-sensing molecule model based on the proton transfer signaling mode. In water-containing medium, the presence of acid can easily induce the proton transfer to the basic H-bond acceptor moiety, which modulates the internal charge transfer state of bis(indolyl)-methene and gives rise to dramatic color changes. As a pH-responsive colorimetric sensor for amino acids, the receptor shows high selectivity and sensitivity for Asp and Glu.
